# Aquathermolytic Upgrading of Zarafshanian Extra Heavy Oil Using Ammonium Alum

**DOI:** 10.3390/molecules30143013

**Published:** 2025-07-18

**Authors:** Amirjon Ali Akhunov, Firdavs Aliev, Nurali Mukhamadiev, Oscar Facknwie Kahwir, Alexey Dengaev, Mohammed Yasin Majeed, Mustafa Esmaeel, Abdulvahhab Al-Qaz, Oybek Mirzaev, Alexey Vakhin

**Affiliations:** 1 Department of Chemistry, Samarkand State University, Samarkand 140104, Uzbekistanm_nurali@mail.ru (N.M.); 2 Institute of Geology and Petroleum Technologies, Kazan Federal University, Kazan 420008, Russiammadzhid@stud.kpfu.ru (M.Y.M.); mustafa.epm@gmail.com (M.E.); 2021alqaz@gmail.com (A.A.-Q.); vahin-a_v@mail.ru (A.V.); 3 Department of Petroleum Exploitation and Reservoir Engineering, National University of Oil and Gas (Gubkin University), Moscow 119991, Russia; dengaev.a@gubkin.ru; 4 Department of Oil and Oil and Gas Processing Technology, Fergana State Technical University, Fergana 150100, Uzbekistan

**Keywords:** heavy oil, in situ upgrading, double salts, ammonium alum, steam injection, thermal enhanced oil recovery, aquathermolysis

## Abstract

The growing global demand for energy necessitates the efficient utilization of unconventional petroleum resources, particularly heavy oil reserves. However, extracting, transporting, and processing these resources remain challenging due to their low mobility, low API gravity, and significant concentrations of resins, asphaltenes, heteroatoms, and metals. In recent years, various in situ upgrading techniques have been explored to enhance heavy oil quality, with catalytic aquathermolysis emerging as a promising approach. The effectiveness of this process largely depends on the development of cost-effective, environmentally friendly catalysts. This study investigates the upgrading performance of water-soluble ammonium alum, (NH_4_)Al(SO_4_)_2_·12H_2_O, for an extra-heavy oil sample from the Zarafshan Depression, located along the Tajikistan–Uzbekistan border. Comprehensive analyses demonstrate that the catalyst facilitates the breakdown of heavy oil components, particularly resins and asphaltenes, into lighter fractions. As a result, oil viscosity was significantly reduced by 94%, while sulfur content decreased from 896 ppm to 312 ppm. Furthermore, thermogravimetric (TG-DTG) analysis, coupled with Fourier-transform infrared spectroscopy (FT-IR), scanning electron microscopy (SEM), and X-ray diffraction (XRD), revealed that the thermal decomposition of ammonium alum produces catalytically active Al_2_O_3_ nanoparticles. These findings suggest that ammonium alum is a highly effective water-soluble pre-catalyst for hydrothermal upgrading, offering a viable and sustainable solution for the development of extra-heavy oil fields.

## 1. Introduction

The world energy demand continues to rise due to population growth, industrial expansion, and urbanization. Despite the global shift toward renewable green energy sources like solar, wind, and hydropower, fossil fuels still dominate in the total world energy balance [1]. Among fossil fuels, heavy and extra-heavy crude oils are one of the most promising energy sources due to their abundant global reserves. As an alternative to conventional hydrocarbons, heavy and extra-heavy oils are expected to play a significant role in the global energy balance in the near future [2]. However, its production, transportation, and processing present considerable challenges. The high viscosity, density, and sulfur content, along with a low hydrogen-to-carbon (H/C) ratio, make extraction and refining more complex and costly. Additionally, the high metal content in heavy oil tends to poison catalysts in conventional reactor systems, reducing processing efficiency and overall feasibility. In the given context, in situ upgrading technologies are highly attractive for heavy oil recovery, as they enhance oil quality directly within the reservoir. Geological structures with impermeable caprocks serve as natural, cost-effective reactors with minimal environmental impact, enabling heavy oil upgrading under suitable thermobaric conditions. Steam-based Enhanced Oil Recovery (EOR) is the dominant method for heavy and extra-heavy crude oils recovery, accounting for over 70% of global production [3]. In Canada alone, Steam-Assisted Gravity Drainage (SAGD) and Cyclic Steam Stimulation (CSS) produce approximately 1.5 million barrels of heavy oil per day [4]. Similarly, in Venezuela’s Orinoco Belt, CSS contributes to over 1 million barrels per day, while in the United States, steam flooding in fields such as Kern River and Midway-Sunset yields over 150,000 barrels per day [5,6,7]. China also extensively applies steam injection in the Liaohe and Xinjiang oil fields, producing several hundred thousand barrels daily [8]. Despite its widespread use, steam-based EOR primarily functions by increasing the mobility of heavy oil in porous media through heat application, facilitating easier extraction. However, once the steam condenses, viscosity can regress to or even exceed initial values, posing significant challenges in transportation and downstream processing. These limitations underscore the need for advanced upgrading strategies and chemical enhancements to sustain production efficiency and improve the economic viability of heavy oil recovery. Introduction of catalysts in steam injection techniques significantly enhances the chemical reactions between steam and native crude oil, facilitating in situ upgrading and improved oil recovery. This approach was first proposed by Hyne in 1986 and has since been extensively studied [9]. Researchers have explored various catalytic systems and their precursors, including water-soluble, oil-soluble, and amphiphilic ones. However, water-soluble catalysts have several advantages over oil-soluble catalysts in aquathermolysis reactions carried out in porous media. First of all metal salts are dispersed more evenly in the steam phase, leading to better contact with the heavy oil components, while oil-soluble pre-catalysts have limited solubility and diffusion, and require additional solvent to achieve the dispersion and ease of delivering the catalysts into the pay zone [10,11]. The water-soluble catalysts are more environmentally friendly than oil-soluble organometallic catalysts as the latter can introduce organic pollutants complicating waste water treatment [12,13]. Finally, water-soluble catalysts are often cheaper and easier to handle compared to complex oil-soluble catalysts, which may require special formulations, solvents, or stabilizers [14,15,16].

Clark et al. was among the first who showed the efficiency of the water-soluble transition metal salts such as iron (II) sulfate heptahydrate and ruthenium (III) chloride in aquathermolytic upgrading of heavy oil samples [17,18,19]. The authors suggest that the iron catalyst facilitated hydrogen transfer between organic molecules, whereas ruthenium exhibited greater selectivity for breaking carbon–heteroatom bonds. Recently, Chen et al. synthesized various water-soluble Fe(III) complexes and employed them to catalyze the aquathermolysis of heavy oil [20]. The results demonstrated a significant viscosity reduction of up to 88.22% under optimal conditions, highlighting the effectiveness of Fe(III) complexes in enhancing heavy oil recovery. Other transition metal salts such as iron, nickel, molybdenum, and cobalt compounds were widely used in the literature to promote aquathermolysis reactions [21,22]. While the role of transition metals (Ni, Fe, Co, Cr, etc.) in promoting aquathermolysis reactions is well-established, relatively few studies have investigated the efficiency of aluminum oxides in this context [17,23,24].

Husein et al. studied the catalytic performance of alumina nanoparticles, which were obtained in situ, on thermal cracking of heavy oil [25]. The presence of Al_2_O_3_ nanoparticles led to a significant pressure buildup and enhanced gas evolution, with H_2_ and CH_4_ identified as major gaseous products, although particle agglomeration at 350 °C introduced uncertainties in the measurements. Viscosity measurements revealed a notable reduction due to thermal treatment, with an overall increase in API gravity observed in the presence of Al_2_O_3_ nanoparticles. However, the simultaneous presence of water and nanoparticles did not result in a statistically significant viscosity change, likely due to cross-linking effects among oil constituents [25]. Nassar et al. investigated the influence of alumina surface acidity on asphaltene adsorption and subsequent oxidation by comparing three alumina samples with similar textural characteristics but varying acid-base properties [26]. Their results showed that asphaltene adsorption increased with the acidity of the alumina, with the most acidic sample exhibiting the highest adsorption capacity. Conversely, the alumina with basic surface properties demonstrated superior catalytic performance during asphaltene oxidation. Notably, they identified a clear relationship between the Freundlich affinity constant and catalytic activity, revealing that higher adsorption affinity enhances the catalytic effectiveness of the adsorbent [26]. Simao et al. classify aluminum as a monometallic post-transition metal and include it among unsupported polymetallic catalysts utilized for heavy oil upgrading [27]. Alumina (Al_2_O_3_) is widely employed in petroleum refining as a catalyst or catalyst support due to its high surface area, thermal stability, and the presence of both acidic and basic sites. Rana et al. examined the influence of alumina supports with varying textural properties on the hydrodesulfurization and hydrodemetallization of Maya crude oil [28]. Additionally, alumina incorporation enhances the catalyst’s resistance to severe deactivation conditions [29]. Aluminum oxides are also integral components of zeolite-based catalysts, which are industrially utilized in Fluid Catalytic Cracking (FCC) of heavy oils [30]. Similarly, Cardona et al. evaluated the effect of alumina doped with Ni nanofluid on the upgrading performance of extra-heavy crude oil and oil recovery factor [31]. The in-depth study of the catalytic role of alumina in petroleum chemistry and its contributions during downstream thermal processes have been extensively discussed in our previous review paper [32] and recent experimental article using different ligands—acetates [33]. For example, Clark et al. [17] investigated the interaction between sulfur-containing compounds in heavy oil and aqueous transition metal ions, including Al^3+^. The study found that Al^3+^ exhibited a 57% selectivity for converting benzothiophene. In comparison, metal ions from platinum group metals demonstrated significantly higher catalytic activity, achieving over 90% benzothiophene conversion. These findings suggest that water-soluble metal salts, including Al^3+^, can significantly enhance aquathermolysis reactions. In another study, Lakhova et al. carried out aquathermolysis of biodegraded heavy crude in the presence of Fe_2_O_3_, Al_2_O_3_, ZnO, and bimetallic catalysts based on Fe and Ni [24]. The comparison revealed that Al_2_O_3_, with a particle size of 40 nm, exhibited the highest performance in reducing resins and asphaltenes, with a reduction degree of 53% and 58%, respectively, compared to the blank sample. Furthermore, Al_2_O_3_ contributed to a reduction in sulfur content from 2.8 wt.% to 2.0 wt.%.

Despite alumina’s well-established role as a catalyst and support in downstream petroleum refining, its application under upstream conditions, particularly during aquathermolysis, remains insufficiently investigated. While previous studies have shown that alumina nanoparticles can promote gas evolution and reduce viscosity during thermal cracking, the structural and molecular transformations of heavy oil constituents resulting from alumina-assisted upgrading have not been systematically examined. Furthermore, the thermal stability and reusability of alumina catalysts under the harsh conditions typical of aquathermolysis remain largely unassessed, constraining their practical implementation in field operations. To address these knowledge gaps, the present study investigates the catalytic performance of alumina during the aquathermolytic upgrading of extra-heavy crude oil from the Zarafshan Depression. In addition, the thermal decomposition behavior of ammonium alum and the characterization of the catalyst post-upgrading are examined to evaluate stability and effectiveness under simulated reservoir conditions.

## 2. Results and Discussions

### 2.1. Thermal Decomposition Pathway of Ammonium Alum

Understanding the thermal decomposition of the pre-catalyst is essential for determining the activation energy required to convert it into its active form. Without the necessary decomposition steps, the catalyst would remain inactive, thus significantly reducing its efficiency. The thermal breakdown process enables the pre-catalyst to evolve into a structure that maximizes its catalytic potential. In the context of aquathermolysis, this often involves the formation of metallic particles or metal oxides, which actively participate in breaking down heavy oils or bitumen into lighter hydrocarbons. Moreover, TG-DTG analysis plays a critical role in evaluating the thermal stability of the pre-catalyst under aquathermolysis reaction conditions. The decomposition behavior of the catalyst precursor has a significant impact on the distribution of products, thereby enabling better control over product yield and quality.

**Figure 1:**
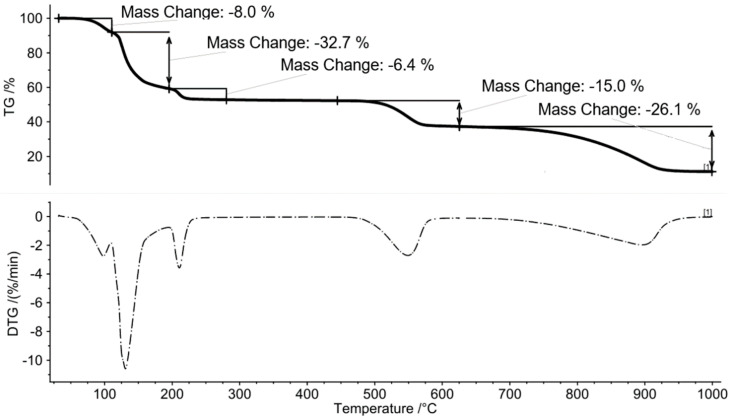
The TGA of (NH_4_)Al(SO_4_)_2_·12H_2_O).

The results of the TGA of ammonium alum (Al(NH_4_)SO_4_·12H_2_O) at a heating rate of 10 K/min are presented in Figure 1. This analysis was performed under a dynamic nitrogen atmosphere, simulating the inert conditions typical during in situ aquathermolysis. The TG-DTG curves indicate that ammonium alum decomposes in three distinct stages. The first stage, occurring between room temperature and 250 °C, is attributed to the evaporation of water. This stage is reflected in the TGA curve (Figure 1) by three peaks, with maxima at 98.22 °C, 128.34 °C, and 216.79 °C. The mass loss associated with dehydration in this stage is approximately 47.4%, which is consistent with the mass loss predicted from the stoichiometry of the dehydration reaction (Equation (1)).

Al(NH_4_)SO_4_ · 12H_2_O → Al(NH_4_)SO_4_ + 12H_2_O (g) (1)

The second stage begins with the decomposition of ammonium sulfate (Al(NH_4_)SO_4_) at approximately 450 °C, with the maximum peak observed at 540.91 °C. This stage ends around 600 °C. FT-IR spectroscopy confirmed the release of ammonia (NH_3_) and sulfur dioxide (SO_2_), supporting the proposed decomposition reaction of ammonium sulfate (Equation (2)):

2[(NH_4_)Al(SO_4_)_2_] → 2NH_3_ + (SO_4_)_3_Al_2_ + SO_2_ + H_2_O + 0.5 O_2_ (2)

**Figure 2:**
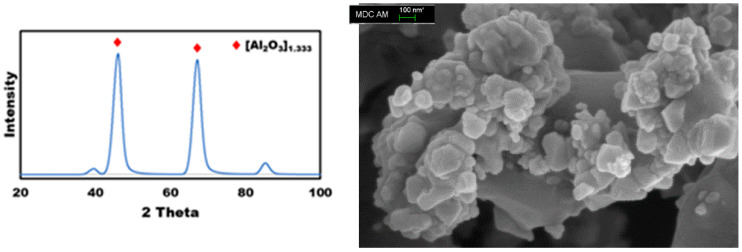
XRD pattern (**left**) and SEM image (**right**) of ammonium alum isolated after upgrading of heavy oil.

The stoichiometric estimation predicts a mass loss of 14.6%, while the TGA-based mass loss was 14.8%, demonstrating excellent agreement between the two methods and confirming the reliability of the data interpretation. The final stage corresponds to the formation of aluminum oxides from the decomposition of aluminum sulfate. This transformation is evidenced by the XRD pattern of the thermolysis residue (Figure 2). The decomposition of aluminum sulfate begins at temperatures above 650 °C, as shown by the following chemical reaction (Equation (3)):

(SO_4_)_3_Al_2_ → Al_2_O_3_ + 3SO_2_ + 3/2 O_2_ (3)

The X-ray diffraction (XRD) pattern of the thermolysis residue is shown in Figure 2 (left). Based on the ICDD reference code 01-075-0921, the only crystalline phase identified corresponds to the γ-Al_2_O_3_, supporting the reaction pathway proposed in Equation (3). The size of the particles after the thermal decomposition of (NH_4_)Al(SO_4_)_2_·12H_2_O) exhibited a nanoscale range with less than 100 nm, as illustrated in Figure 2 (right). This nanorange particle size confers several advantages, including a high surface area, enhanced molecular diffusion, increased resistance to coke formation, and improved thermal stability.

### 2.2. Upgrading Performance of Ammonium Alum

#### 2.2.1. Viscosity of Upgraded Oil

**Figure 3:**
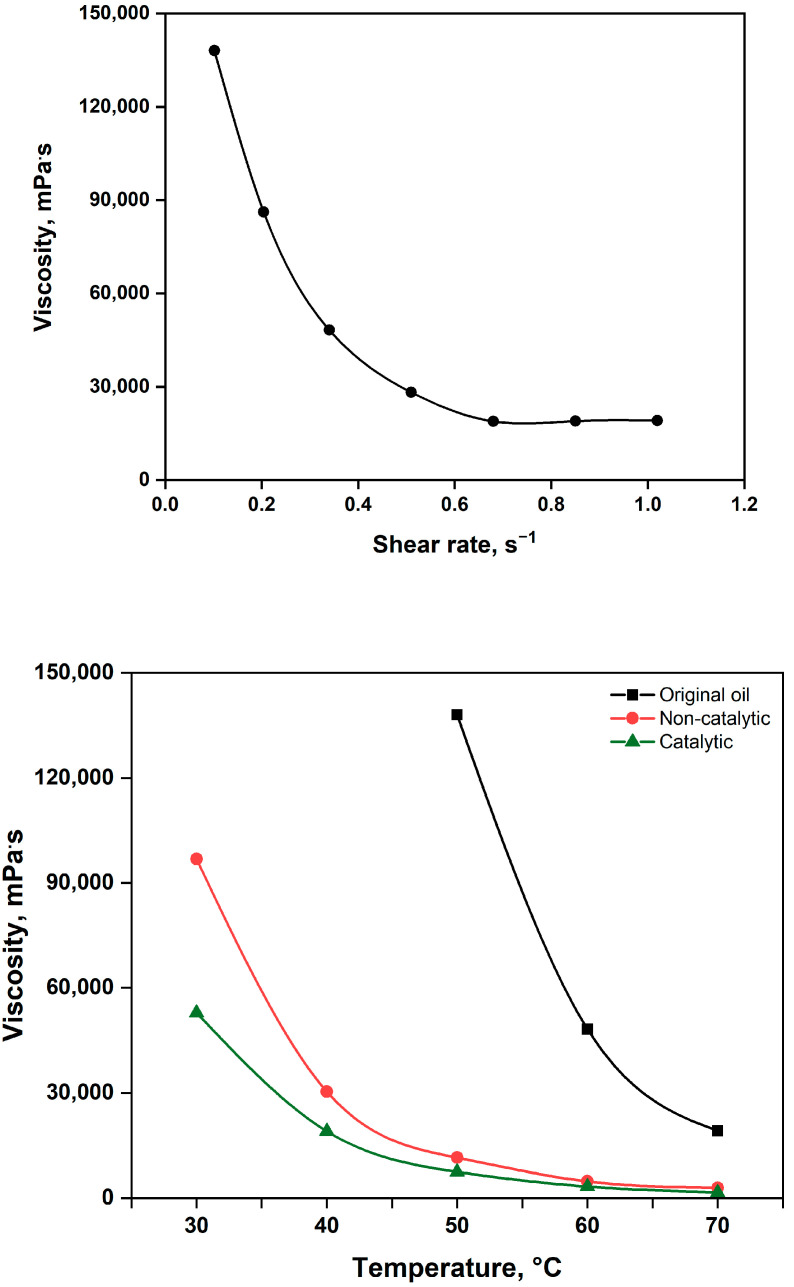
Shear rate (**up**) and temperature (**down**) dependent viscosity values.

The shear rate-dependent viscosity of the original extra-heavy oil exhibited non-Newtonian flow behavior, decreasing markedly from 138.059 mPa·s to 18.898 mPa·s as the shear rate increased from 0.1 s^−1^ to 1.02 s^−1^ (Figure 3, up). Throughout the manuscript, “original oil” refers to the crude oil prior to any hydrothermal upgrading treatment, while the “non-catalytic” sample corresponds to the crude oil after hydrothermal upgrading conducted without the addition of the catalyst. Non-catalytic upgrading reduced the viscosity to 11 490 mPa·s. With the addition of catalyst particles (Catalytic), viscosity was reduced even more significantly, reaching 7 461 mPa·s measured under the same conditions (Figure 3, down). This substantial reduction is attributed to changes in the chemical composition of the oil. The results of SARA (Saturates, Aromatics, Resins, and Asphaltenes) analysis and their correlation with viscosity are discussed in Section 2.2.2. Notably, molecular isomerization and structural reorganization of heavy fractions during aquathermolysis also contribute to viscosity reduction [34,35,36]. The catalytic effect primarily lies in accelerating the destructive hydrogenation of large hydrocarbon molecules, particularly by cleaving weak bonds such as C–S, C–N, and C–O. Additionally, catalyst particles promote the water–gas shift reaction, generating hydrogen species that stabilize hydrocracked fragments of resins and asphaltenes [37,38,39].

**Figure 4:**
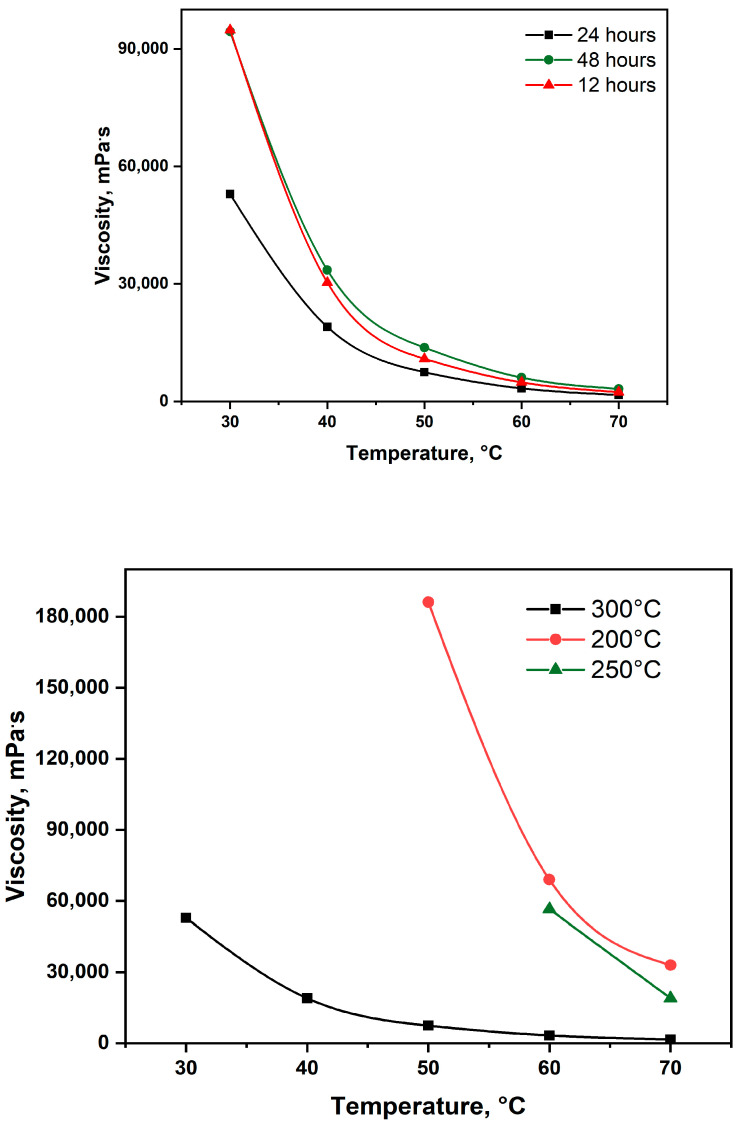
The influence of upgrading duration (**up**) and temperature (**down**) on the viscosity values.

Three different catalyst residence times at aquathermolysis temperature of 300 °C—12, 24, and 48 h—were investigated to evaluate their effect on viscosity reduction (Figure 4, up). Among these, the 24 h residence time resulted in the most significant decrease in viscosity. A shorter residence time of 12 h was likely insufficient for completing the destructive hydrogenation reactions, leaving heavier hydrocarbon fragments partially unconverted and thus limiting the extent of viscosity reduction. Conversely, an extended residence time of 48 h led to overcracking, whereby valuable middle distillates were further degraded into gaseous products. Furthermore, prolonged exposure may have contributed to catalyst deactivation, which not only diminishes catalytic activity but also promotes undesired side reactions, including polymerization, particularly in the absence of sufficient hydrogen species [38,40].

The influence of the temperature on the upgrading performance of the (NH_4_)Al(SO_4_)_2_·12H_2_O) was evaluated with the optimum residence time—24 h. Three temperature ranges (200 °C, 250 °C and 300 °C) within the aquathermolysis window were considered, and generally speaking viscosity has been reduced with increasing the upgrading temperature (Figure 4, down). The incremental viscosity reduction by increasing the temperature from 200 °C to 250 °C was much lower than incremental viscosity reduction within the 250 °C–300 °C temperature range.

#### 2.2.2. Group-Chemical Composition of Upgraded Oil

**Figure 5:**
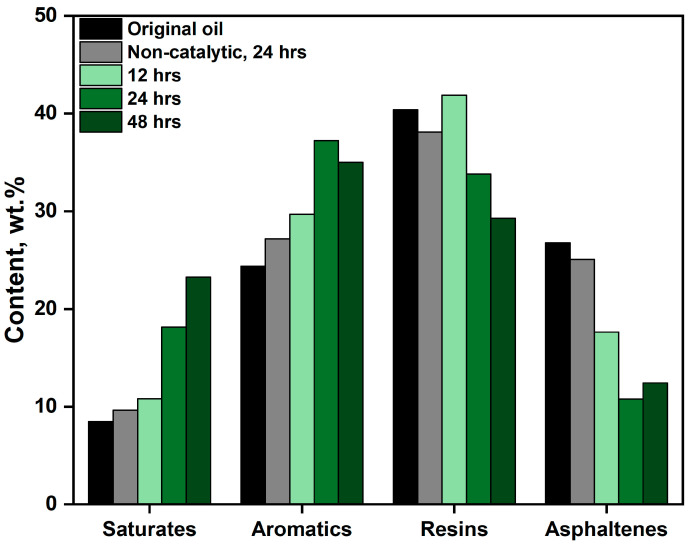
Distribution of SARA fractions under varying catalyst residence times.

Fractionation of crude oil into its SARA (Saturates, Aromatics, Resins, Asphaltenes) constituents provides critical insight into the extent of upgrading performance of the processes. Improved oil quality is typically characterized by an increased proportion of low molecular weight hydrocarbons—namely saturates and aromatics—and a corresponding reduction in high molecular weight species such as resins and asphaltenes. In this study, SARA analysis was employed to evaluate compositional changes in extra-heavy crude oil subjected to both non-catalytic and catalytic aquathermolysis, compared to the original sample. Additionally, isolation of light fractions (saturates and aromatics) enabled detailed structural analysis via Gas Chromatography–Mass Spectrometry (GC-MS), facilitating identification of destructive hydrogenation products. The evolution of SARA fractions under varying catalyst residence times is presented in Figure 5. Under non-catalytic aquathermolysis at 300 °C for 24 h, only a modest reduction in resin and asphaltene content was observed, with a slight increase in the lighter saturate and aromatic fractions, attributed to thermal cracking and partial hydrogenation. In contrast, the presence of catalytic nanoparticles significantly enhanced the upgrading process. After 24 h of catalytic treatment, the asphaltene content decreased from 25.1 wt.% (blank, no catalyst) to 10.8 wt.% (Figure 5), while the saturate fraction doubled. Overall, a clear trend was observed: with increasing catalytic residence time, the saturate content progressively increased, while resins and asphaltenes declined accordingly. The most effective upgrading outcome was achieved at a 24 h catalytic residence time, where the asphaltene content dropped from 26.8 wt.% (original sample) and 25.1 wt.% (blank) to 10.8 wt.%, while the saturate content rose from 8.5 wt.% and 9.6 wt.% to 18.1 wt.%, respectively. These changes suggest extensive destructive hydrogenation of high molecular weight fractions into more mobile, lower molecular weight compounds. However, extending the catalytic upgrading beyond 24 h led to adverse effects. The accumulation of resin destruction products, coupled with limited hydrogen availability, favored polymerization reactions, thereby increasing the asphaltene content above that observed at the 24 h mark. This recombination of free radicals into heavier structures is likely due to insufficient hydrogen ion concentration to stabilize the reactive intermediates—a trend corroborated by the data on free radical abundance. These compositional changes are consistent with viscosity measurements (Figure 4, up), reinforcing the correlation between molecular weight distribution and flow properties in upgraded oil. Hence, a 24 h catalytic treatment appears to represent the optimal balance between effective upgrading and minimization of secondary polymerization reactions. The selection of temperature ranges in heavy oil upgrading processes is critical, as key parameters—such as reaction kinetics, the generation of lighter hydrocarbon fractions, and compositional structural transformations—are highly temperature-dependent.

**Figure 6:**
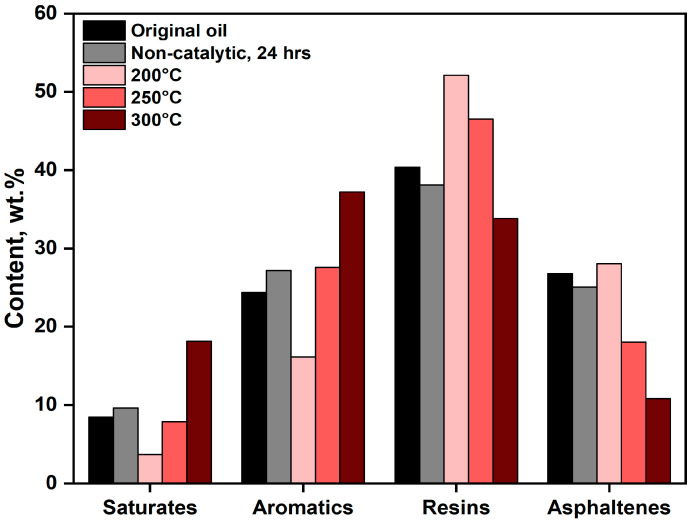
Distribution of SARA fractions under varying upgrading temperature ranges.

As the upgrading temperature increases, the proportions of saturates and aromatics rise correspondingly, while the concentrations of resins and asphaltenes decline (Figure 6). Notably, the group composition of extra-heavy crude oil subjected to non-catalytic upgrading at 300 °C closely resembles that of oil upgraded catalytically at 250 °C (Figure 6), indicating a potential equivalence in product quality achieved through different processing conditions.

#### 2.2.3. Sulfur Content of Upgraded Oil

**Figure 7:**
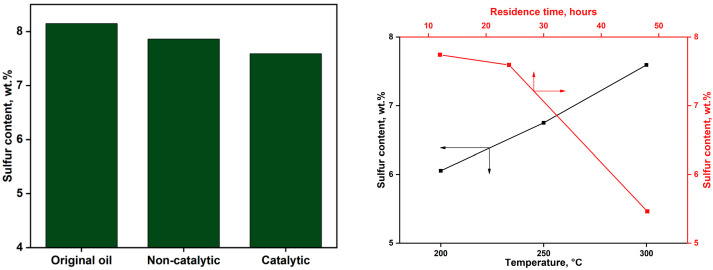
The content of sulfur before and after non-catalytic and catalytic upgrading (**left**), and its dependency on the catalyst’s residence times (red curve) and reaction temperatures (black curve; **right**).

Sulfur is the third most abundant element in petroleum, following carbon and hydrogen, with concentrations in crude oil ranging from 0.01 to 7.69 wt.% [41]. Sulfur exists in both inorganic and organic forms. Inorganic sulfur species—such as elemental sulfur, hydrogen sulfide (H_2_S), and pyrite—may occur in either dissolved or suspended phases [42]. However, the predominant form of sulfur in crude oil is organic, comprising compounds such as thiols, sulfides, and thiophenes. During aquathermolysis, the reaction mechanism often initiates with the cleavage of thermodynamically weak sulfur-containing bonds, such as carbon–sulfur (C–S) linkages, which are prevalent in extra-heavy oils. Consequently, the sulfur content is considered a key indicator of aquathermolysis efficiency: a greater reduction in sulfur concentration after upgrading typically reflects a higher degree of conversion. Figure 7 (left) illustrates the sulfur content in extra-heavy crude oil samples before and after both non-catalytic and catalytic upgrading processes. The impact of catalysts’ residence times and catalytic reaction temperature parameters are also evaluated.

Non-catalytic upgrading at 300 °C for 24 h resulted in a modest reduction in sulfur content, decreasing from 8.15 wt.% to 7.86 wt.%. The incorporation of ammonium alum as an additive further enhanced desulfurization, lowering the sulfur content to 7.59 wt.%. This additional reduction is likely attributable to the promotion of hydrodesulfurization reactions facilitated by the presence of the alum. Figure 7 (right) illustrates the dependency of sulfur content on both the catalyst residence time (red curve) and the reaction temperature (black curve). The results show that extending the catalyst residence time from 12 to 48 h significantly decreases the sulfur content from 7.74 wt.% to 5.46 wt.%, demonstrating that prolonged exposure allows more thorough cleavage of sulfur-containing bonds. Conversely, lowering the reaction temperature from 300 °C to 200 °C also leads to a notable reduction in sulfur content, from 7.59 wt.% at 300 °C to 6.05 wt.% at 200 °C. This trend indicates that, under the tested conditions, lower temperatures favor the desulfurization process, possibly by suppressing secondary reactions that stabilize sulfur compounds.

#### 2.2.4. GC-MS of Saturates and Aromatics of Upgraded Oil

**Figure 8:**
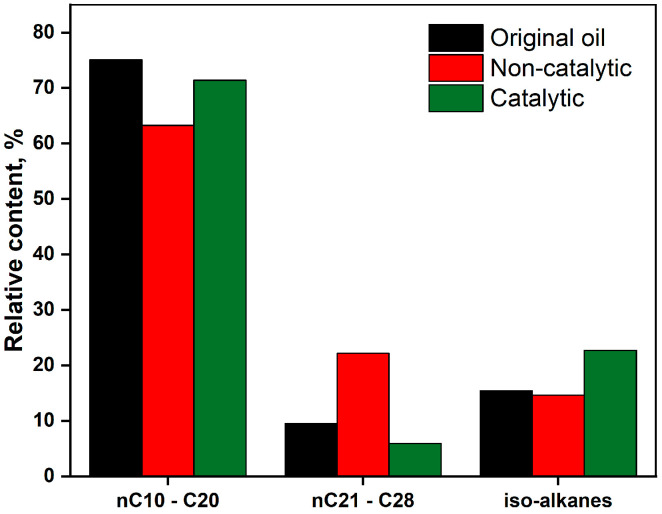
Distribution of nC10–C20, nC21–C28, and iso-alkanes based on GC-MS spectra after non-catalytic and catalytic upgrading at 300 °C for 24 h with concentration of 0.2%wt. by metal.

**Figure 9:**
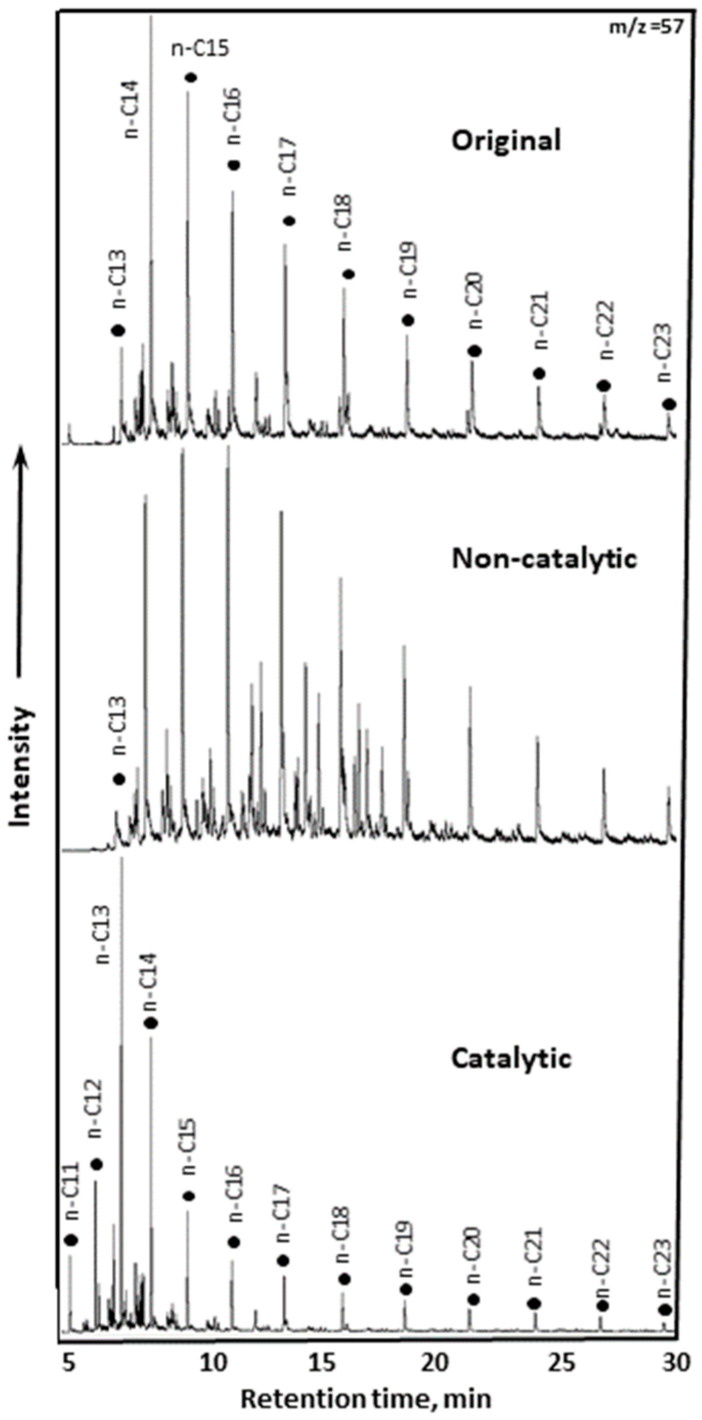
The spectra of saturates as per *m*/*z* = 57.

The distribution of saturates—nC10–C20, nC21–C28, and iso-alkanes in extra-heavy oil before and after catalytic and non-catalytic treatment is presented in Figure 8, and a spectra of saturates as per *m*/*z* = 57 are illustrated in Figure 9. The catalytic system demonstrates superior performance by increasing light and branched hydrocarbons while reducing heavier fractions—this translates to improved oil quality, reduced viscosity, and enhanced flow behavior. The relative abundance of light n-alkanes is largely preserved in the catalytic process, while it decreases in non-catalytic treatment. This suggests that the (NH_4_)Al(SO_4_)_2_·12H_2_O helps stabilize or regenerate light alkanes, possibly through the cracking of heavier fractions and hydrogenation reactions. Non-catalytic conditions likely lead to partial degradation due to uncontrolled thermal effects, which led to the accumulation of nC21–C28 alkanes. This likely occurred because thermal treatment without catalysis resulted in partial cracking of heavier molecules without subsequent hydrogenation, producing intermediate-weight hydrocarbons. In contrast, the catalytic route reduces the heavy alkane fraction, indicating more effective cracking and conversion into lighter fractions and into iso-alkanes, which improves oil quality.

The relative content of iso-alkanes is notably increased with the addition of the catalyst particles from 15% to 23%. The presence of the elevated branched alkanes in the composition of oil not only lowers pour point due to isomerization, but also improves combustion properties of gasoline fractions such as octane number. Thus, the catalyst reconfigured molecular structure, which improved the overall mobility.

#### 2.2.5. Electron Paramagnetic Resonance (EPR) Spectroscopy of Upgraded Extra-Heavy Oil

**Figure 10:**
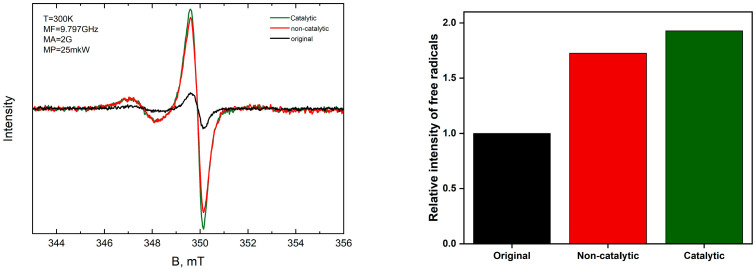
EPR (**left**) spectra and (**right**) relative intensity of the free radical lines in asphaltenes isolated from the original, non-catalytic, and catalytic extra-heavy crude oil samples.

EPR spectroscopy is a highly sensitive analytical technique widely used for the investigation of species containing unpaired electrons, including free radicals and other paramagnetic entities [43,44]. In the present study, EPR analysis was employed to quantify the concentration of free radicals within asphaltenes isolated from extra-heavy crude oil before and after upgrading in the absence and presence of the catalyst, which serves as an indicator of the aquathermolytic cleavage of alkyl substitutes from the asphaltene cores with unpaired electrons and aromaticity characteristics inherent to its complex structures. The EPR spectra and relative intensity of free radicals estimated from the spectra are illustrated in Figure 10.

The intensity of the free radical signal associated with asphaltenes precipitated from the original extra-heavy crude oil is normalized to a value of 1, serving as the baseline. All other free radical line intensities are then compared relative to this reference. The relative intensity of free radicals increases significantly after non-catalytic upgrading, approximately 1.7 times higher than the original. The intensity of free radicals increases owing to the aquathermolytic bond cleavage or structural rearrangements of asphaltene fragments, which lead to the increase in the number of unpaid electrons within the asphaltenes. Both cases improve the mobility of extra-heavy crude oil. After catalytic upgrading, the highest relative intensity of free radical line was observed—almost two times of original sample. This implies that the catalytic process further promotes aquathermolysis reactions.

#### 2.2.6. Gaseous Products Evolved After Upgrading of Extra-Heavy Oil

**Figure 11:**
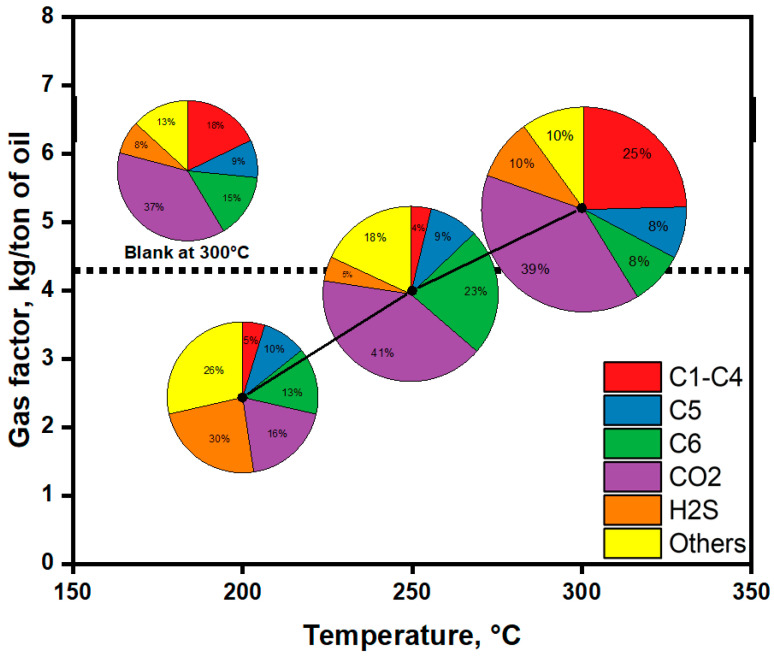
The gas factor and composition of the evolved gases after upgrading of extra-heavy oil at different temperatures.

Gas chromatography results depicted in Figure 11 offer vital insights into the interplay between temperature and catalytic influence on the gas yield and composition during aquathermolytic upgrading of extra-heavy oil. The figure presents both the quantitative gas factor (kg of gas per ton of oil) and qualitative composition via pie charts, comparing non-catalytic conditions (blank at 300 °C) with catalytic conditions at 200 °C, 250 °C, and 300 °C, each over a reaction time of 24 h. A pronounced positive correlation is observed between reaction temperature and gas yield under catalytic conditions. The gas factor increases steadily from 2.4 kg/ton at 200 °C to 5.2 kg/ton at 300 °C, evidencing intensified thermal decomposition and gas evolution at elevated temperatures. This trend underscores the enhanced aquathermolytic cracking mechanisms facilitated by higher thermal energy. Notably, the gas factor at 300 °C in the catalytic run surpasses the non-catalytic reference (5.2 vs. 4.2 kg/ton), clearly illustrating the catalyst’s role in accelerating bond cleavage reactions by lowering activation energies. CO_2_ remains the dominant gas component across all catalytic temperatures, with its share ranging from 16% at 200 °C to 41% at 250 °C. This is attributed to the water–gas shift reaction (CO + H_2_O → CO_2_ + H_2_), which becomes increasingly active under hydrothermal conditions [45,46,47]. Hydrogen sulfide (H_2_S) content is consistently high (ranging from 26% at 200 °C to 10% at 300 °C), which correlates with the significant sulfur content in the crude oil (see Figure 7). The progressive decline in H_2_S with increasing temperature may suggest secondary reactions or conversion of sulfur species at higher thermal regimes.

A significant finding is the overall increase in light hydrocarbons (C1–C4) with rising temperature under catalytic conditions, although a slight decrease is observed at 250 °C due to the preferential formation of C6 hydrocarbons at this intermediate temperature. This surge confirms that aquathermolysis facilitates alkyl chain cleavage, especially at higher temperatures. Comparatively, the blank (non-catalytic) sample at 300 °C produces only 18% C1–C4, further reinforcing the catalytic enhancement of hydrocarbon gas evolution. The catalytic influence is particularly evident at 300 °C, where both gas quantity and favorable composition (e.g., higher C1–C4 and CO_2_, reduced H_2_S) are optimized. The combined effect of enhanced hydrocracking, water–gas shift dynamics, and sulfur removal pathways underpins the superior performance of the ammonium alum in transforming heavy fractions into more valuable products.

## 3. Materials and Methods

### 3.1. Materials

An extra-heavy crude oil sample sourced from the Yangi Uzbekiston field, located at a depth of 400–750 m within the Zarafshan petroleum province, which lies on the border between Tajikistan and Uzbekistan. The sample was provided by Samarkand State University, and its key parameters are outlined in Table 1.

(NH_4_)Al(SO_4_)_2_·12H_2_O, aluminum oxide, as well as other organic solvents such as n-hexane, toluene, chloroform, and methyl alcohol were obtained from Sigma-Aldrich. (Burlington, MA, USA)

### 3.2. Catalytic Performance of Ammonium Alum

**Figure 12:**
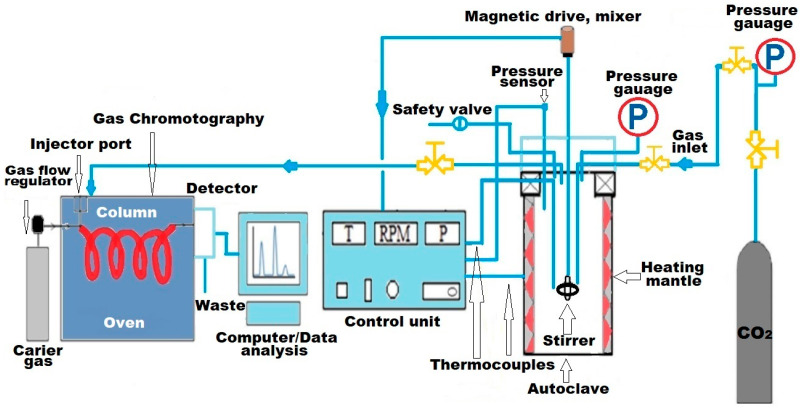
Schematic illustration of HP-HT reactor coupled with GC. Adapted from [48].

The non-catalytic and catalytic upgrading of heavy oil samples was conducted in a High Temperature–High Pressure (HT-HP) reactor, equipped with a stirrer and manufactured by Par Instruments (Moline, IL, USA), with a volume capacity of 300 mL. The experimental system consisted of a crude oil and water mixture, with a mass ratio of 70:30. In the catalytic upgrading tests, the concentration of ammonium alum ((NH_4_)Al(SO_4_)_2_·12H_2_O) was adjusted to 0.2% by weight, based on the metal concentration in the heavy oil. Prior to the experiment, the batch reactor was purged with nitrogen for 15 min to eliminate air from the system. Nitrogen was then injected into the reactor to a pressure of 10 bar after sealing the exhaust valve. A leakage check was performed for 5 min before heating began. The reactor was then heated to a temperature range of 200–300 °C at a rate of 4 °C per minute. Isothermal heating was applied for durations of 12, 24, and 48 h. A schematic representation of the HP-HT reactor setup, coupled with gas chromatography (GC), is shown in Figure 12.

Following the upgrading process, the autoclave was allowed to cool under room temperature, and the pressure and temperature values were recorded immediately prior to the gas measurement procedure, as detailed in Section 3.3.1. These temperature-dependent pressure readings are essential for applying the Mendeley–Clapeyron equations, which are used to estimate the total amount of evolved gaseous products. The water present in the oil phase was separated by centrifugation using an Eppendorf 5804R (Eppendorf AG, Hamburg, Germany) at 40 °C and 3000 rpm. The upgraded oil was then analyzed using various analytical techniques. The isolated catalyst particles were subsequently characterized using Scanning Electron Microscopy (SEM) (Carl Zeiss Microscopy GmbH, Oberkochen, Germany), Thermogravimetric (TG) analysis (Netzsch-Gerätebau GmbH, Selb, Germany), and X-ray Diffraction (XRD) (Rigaku Corporation, Tokyo, Japan) techniques.

### 3.3. Analytical Methods

#### 3.3.1. Gas Chromatography Analysis

The High Temperature-High Pressure (HT-HP) reactor was integrated with a Gas Chromatography (GC) system, specifically the Crystal 5000 model manufactured by Chromatec (Russia), to separate the evolved gas mixture into its individual components. This separation was performed using a capillary column coated with a stationary phase, measuring 30 m in length and 0.25 mm in diameter. Helium and argon were employed as carrier gases. The temperature program involved heating from 35 °C to 250 °C at a rate of 2 °C/min. The gas flow velocity was maintained at 15 mL/min. 

#### 3.3.2. Viscosity Measurement

Viscosity measurements of the crude oil, both before and after upgrading (with and without catalysts), were conducted using a FUNGILAB Alpha L viscometer (Fungilab, Sant Feliu de Llobregat, Barcelona, Spain) coupled with a HUBER MPC K6 cooling circulator (HUBER, Atlanta, GA, USA). A TL7 spindle was selected based on the viscosity range of the untreated crude oil. Measurements were recorded once torque stabilization was achieved, typically within a range of 50–90%. The instrument’s measurement error was within ±1%.

#### 3.3.3. SARA Fractionation Analysis

The compositional analysis of the crude oil samples before and after the upgrading process was carried out using SARA fractionation, which classifies oil components into saturates, aromatics, resins, and asphaltenes. Asphaltenes were precipitated using a crude oil-to-hexane ratio of 1:40. The remaining maltene fraction was further separated into saturates, aromatics, and resins based on differential solubility. Saturates were extracted using hexane, aromatics with toluene, and resins with a toluene-methanol mixture (1:3). The separation process was performed using a chromatographic column packed with alumina that had been pre-calcined at 420 °C.

#### 3.3.4. Total Sulfur Analysis

The total sulfur content in the extra-heavy crude oil samples was quantitatively analyzed using energy dispersive X-ray fluorescence (EDXRF) spectrometry, performed on a Spectroscan SE instrument manufactured by LLC “Spectron” (St. Petersburg, Russia). To mitigate matrix effects commonly encountered in the analysis of heavy and extra-heavy oil, and compositionally complex structures, the samples were pre-diluted using heavy mineral oil (MOWH—Mineral Oil White Heavy). This dilution step serves to reduce matrix mismatch, improve sample homogeneity, and enhance measurement precision. Despite this precaution, it is acknowledged that the presence of substantial concentrations of heavy metals (e.g., vanadium, nickel) and heteroatoms (e.g., nitrogen, oxygen) in the crude oil matrix may still introduce potential interferences or signal suppression, which could affect the absolute quantification of sulfur. Nevertheless, for the primary purpose of this study—comparing relative changes in sulfur content before and after catalytic aquathermolysis treatments—such systematic deviations are considered consistent across all samples. Therefore, the obtained sulfur concentration values are deemed sufficiently reliable for assessing the upgrading performance and evaluating the degree of desulfurization resulting from catalytic upgrading.

#### 3.3.5. Fourier Transform Infrared (FT-IR) Spectroscopy

The functional groups present in the crude oil samples and their fractions were identified using a Bruker Vector 22 FT-IR spectrometer (Bruker, Billerica, MA, USA) within the spectral range of 4000–600 cm^−1^. This analysis focused on the characteristic absorption peaks corresponding to methyl (D1380) and methylene groups (D720, D1465), aromatics (D1600), oxygen-containing (D1710), and sulfur-containing functional groups (D1030). Structural modifications in the oil matrix were assessed using Fourier-derived parameters (4–8), which are the ratio of optical density value at the maximum of the corresponding absorption bands:

Aromaticity Index (A-Factor) = D1600/D720(4)

Oxidation Index (O-Factor) = D1710/D1465(5)

Branching Index (B-Factor) = D1380/D1465(6)

Aliphaticity Index (P-Factor) = (D720 + D1380)/D1600(7)

Sulfurization Index (S-Factor) = D1030/D1465(8)

#### 3.3.6. Electron Paramagnetic Resonance (EPR) Spectroscopy

EPR spectra were recorded using a Bruker ESP-300 spectrometer operating in the X-band frequency range of 9.4–9.9 GHz. A magnetic field strength between 20 and 1600 mT was applied with a fixed modulation frequency of 100 kHz. Nitrogen gas was used as an inert carrier to maintain sample temperature, injected through a quartz tube heated by a high-temperature resonator (ER 4114HT). The heating program was set from 293 K to 780 K, with temperature increments of 8–15 °C and a heating rate of 2 °C/min. Identical experimental conditions were maintained across all measurements to ensure accurate comparisons of free radical (FR) concentrations. The crude oil sample prior to upgrading was used as a reference, representing the baseline FR concentration.

#### 3.3.7. Gas Chromatography–Mass Spectrometry (GC-MS) Analysis

To further analyze the composition of crude oil before and after upgrading, particularly the saturated and aromatic hydrocarbon fractions, GC-MS analysis was performed using a Chromatec-Crystal 5000 GC system coupled with an ISQ mass spectrometer (Thermo Fisher Scientific, Waltham, MA, USA). The analysis was carried out using a 30 m × 0.25 mm capillary column with helium as the carrier gas at a flow rate of 1 mL/min. The temperature program consisted of an initial ramp from 100 °C to 150 °C at a rate of 3 °C/min, followed by a second ramp from 150 °C to 300 °C at 12 °C/min. Data analysis and compound identification were conducted using Xcalibur software 4.3 and referenced against established spectral databases.

#### 3.3.8. Catalyst Characterization

The physicochemical properties of the catalyst before and after the upgrading process were assessed using multiple analytical techniques:

X-ray Diffraction (XRD). The crystalline phases and structural properties of the isolated catalyst particles were analyzed using X-ray Powder Diffraction (XRD). Data were collected using a Rigaku MiniFlex 600 diffractometer (Rigaku Corporation, Tokyo, Japan) equipped with a D/teX Ultra silicon strip detector. The instrument utilized Cu Kα1 radiation (40 kV, 15 mA), scanning over a 2θ range of 2–100° with a step size of 0.02° and a dwell time of 0.24 s per step.

Scanning Electron Microscopy (SEM) and Energy-Dispersive X-ray Spectroscopy (EDX). The morphology, elemental composition, and particle size distribution of the catalyst particles were examined using a Carl Zeiss Merlin SEM (Carl Zeiss Microscopy GmbH, Oberkochen, Germany) integrated with an Aztec X-Max EDX detector (Oxford Instruments, Abingdon, UK). Samples were gold-coated (~10 nm thickness) using a Quorum Q150 ES (Quorum Technologies Ltd., Laughton, UK) sputter coater. Secondary electron (SE) imaging was performed at 5 kV and 300 pA, while EDX mapping utilized an accelerating voltage of 20 kV and a beam current of 1 nA.

Thermogravimetric Analysis (TGA). The thermal stability and degradation profile of the catalyst were evaluated using a Netzsch TG 209 F1 Libra (Netzsch-Gerätebau GmbH, Selb, Germany) instrument integrated with the FT-IR. Approximately 10 mg of sample was heated from room temperature to 600 °C at a constant heating rate of 10 °C/min under an argon flow of 50 mL/min.

## 4. Conclusions

This study comprehensively evaluated the catalytic performance of water-soluble ammonium alum in the aquathermolytic upgrading of extra-heavy crude oil from the Zarafshan Depression. Thermogravimetric and XRD analyses confirmed that ammonium alum decomposes under aquathermolysis conditions to form catalytically active γ-Al_2_O_3_ nanoparticles, which promote cracking, hydrogen transfer, and sulfur removal pathways during upgrading. These catalytic effects resulted in a dramatic viscosity reduction of up to 94% and a doubling of the saturate fraction, while asphaltenes decreased by over 50% under optimal conditions (300 °C, 24 h). The catalyst also facilitated a 65% reduction in sulfur content and increased the yield of light hydrocarbons (C_1_–C_4_) to 25%, outperforming non-catalytic treatment. These improvements are attributed to enhanced hydrocracking of heavy fractions, water–gas shift reactions, and the effective dispersion of the catalyst within the steam phase, which ensures intimate contact with oil constituents. In summary, ammonium alum demonstrates significant potential as a low-cost, water-soluble, and environmentally benign pre-catalyst for in situ upgrading of extra-heavy oil. Future studies should focus on dynamic core flooding tests, field-scale implementation, and long-term environmental assessments to advance the industrial application of this catalyst system.

## Figures and Tables

**Table 1:** Parameters of extra-heavy crude oil.

Parameters	Values
Viscosity (at 50 °C), mPa·s	138,059
Elemental composition, % wt.	
C	75.92
H	11.22
S	8.15
N	2.54
O *	2.17
H/C_atomic_	1.77
Group composition, % wt.	
Saturates	9
Aromatics	24
Resins	40
Asphaltenes	27

* Estimated value.

## Data Availability

Dataset available on request from the corresponding author.

## References

[1] Hascakir B. (2025). Accelerating the Energy Transition with Carbon Capture, Utilization, and Interdisciplinary Innovation. Pet. Sci. Technol..

[2] Pang X. (2023). Evaluation of the Global Oil and Gas Resources. Quantitative Evaluation of the Whole Petroleum System: Hydrocarbon Thresholds and Their Application.

[3] Seidy-Esfahlan M., Tabatabaei-Nezhad S.A., Khodapanah E. (2024). Comprehensive Review of Enhanced Oil Recovery Strategies for Heavy Oil and Bitumen Reservoirs in Various Countries: Global Perspectives, Challenges, and Solutions. Heliyon.

[4] Chai M., Chai L., Nourozieh H., Chen Z., Yang M. (2024). A Technical, Economic, and Environmental Assessment on Dimethyl Ether (DME) as a Renewable Solvent from Carbon Dioxide Utilization (CCU) for Heavy Oil Recovery: A Real Field in Surmont, Canada as Case Study. Chem. Eng. J..

[5] Rodríguez J.R., Naranjo P.A.L., Molina J.W., Molina J.D., Molina F., Matute A.K. Unveiling Success From Cyclic Steam Injections for Heavy Oil Recovery in India and the Orinoco Oil Belt. Proceedings of the SPE EOR Conference at Oil and Gas West Asia.

[6] Bursell C.G., Pittman G.M. (1975). Performance of Steam Displacement in the Kern River Field. J. Pet. Technol..

[7] Gates C.F., Sklar I. (1971). Combustion as a Primary Recovery Process-Midway Sunset Field. J. Pet. Technol..

[8] Dong X., Liu H., Wu K., Chen Z. EOR Potential in the Post Steam Injection Era: Current and Future Trends. Proceedings of the SPE Improved Oil Recovery Conference.

[9] Hyne J.B. (1986). Aquathermolysis: A Synopsis of Work on the Chemical Reaction between Water (Steam) and Heavy Oil Sands During Simulated Steam Stimulation. https://www.osti.gov/etdeweb/biblio/220464.

[10] Wang J., Liu L., Zhang L., Li Z. (2014). Aquathermolysis of Heavy Crude Oil with Amphiphilic Nickel and Iron Catalysts. Energy Fuels.

[11] Wang L., Guo J., Li C., Xiong R., Chen X., Zhang X. (2024). Advancements and Future Prospects in In-Situ Catalytic Technology for Heavy Oil Reservoirs in China: A Review. Fuel.

[12] Kobayashi S., Manabe K. (2002). Development of Novel Lewis Acid Catalysts for Selective Organic Reactions in Aqueous Media. Acc. Chem. Res..

[13] Hanafi M.F., Sapawe N. (2020). A Review on the Current Techniques and Technologies of Organic Pollutants Removal from Water/Wastewater. Mater. Today Proc..

[14] Suwaid M.A., Varfolomeev M.A., Al-Muntaser A.A., Yuan C., Starshinova V.L., Zinnatullin A., Vagizov F.G., Rakhmatullin I.Z., Emelianov D.A., Chemodanov A.E. (2020). In-Situ Catalytic Upgrading of Heavy Oil Using Oil-Soluble Transition Metal-Based Catalysts. Fuel.

[15] Muraza O., Galadima A. (2015). Aquathermolysis of Heavy Oil: A Review and Perspective on Catalyst Development. Fuel.

[16] Wang Q., Zhang S., Chen X., Ni J., Du J., Li Y., Xin X., Zhao B., Chen G. (2024). Synergistic Catalysis of Water-Soluble Exogenous Catalysts and Reservoir Minerals during the Aquathermolysis of Heavy Oil. Molecules.

[17] Clark P.D., Lesage K.L., Tsang G.T., Hyne J.B. (1988). Reactions of Benzo [b] Thiophene with Aqueous Metal Species: Their Influence on the Production and Processing of Heavy Oils. Energy Fuels.

[18] Clark P.D., Kirk M.J. (1994). Studies on the Upgrading of Bituminous Oils with Water and Transition Metal Catalysts. Energy Fuels.

[19] Clark P.D., Dowling N.I., Hyne J.B., Lesage K.L. (1987). The Chemistry of Organosulphur Compound Types Occurring in Heavy Oils: 4. the High-Temperature Reaction of Thiophene and Tetrahydrothiophene with Aqueous Solutions of Aluminium and First-Row Transition-Metal Cations. Fuel.

[20] Chen S., Zhang S., Feng J., Long X., Hu T., Chen G. (2024). Water-Soluble Fe (III) Complex Catalyzed Coupling Aquathermolysis of Water-Heavy Oil-Methanol. Catalysts.

[21] Aliev F., Kholmurodov T., Mirzayev O., Tajik A., Mukhamadiev N., Slavkina O., Nourgalieva N., Vakhin A. (2023). Enhanced Oil Recovery by In-Reservoir Hydrogenation of Carbon Dioxide Using Na-Fe_3_O_4_. Catalysts.

[22] Khafizov N.R., Madzhidov T.I., Yuan C., Varfolomeev M.A., Kadkin O.N. (2022). Theoretical Insight into the Catalytic Effect of Transition Metal Ions on the Aquathermal Degradation of Heavy Oil: A DFT Study of Cyclohexyl Phenyl Amine Cleavage. Fuel.

[23] Medina O.E., Céspedes S., Zabala R.D., Franco C.A., Pérez-Cadenas A.F., Carrasco-Marín F., Lopera S.H., Cortés F.B., Franco C.A. (2022). A Theoretical and Experimental Approach to the Analysis of Hydrogen Generation and Thermodynamic Behavior in an in Situ Heavy Oil Upgrading Process Using Oil-Based Nanofluids. Catalysts.

[24] Lakhova A., Petrov S., Ibragimova D., Kayukova G., Safiulina A., Shinkarev A., Okekwe R. (2017). Aquathermolysis of Heavy Oil Using Nano Oxides of Metals. J. Pet. Sci. Eng..

[25] Husein M.M., Alkhaldi S.J. (2014). In Situ Preparation of Alumina Nanoparticles in Heavy Oil and Their Thermal Cracking Performance. Energy Fuels.

[26] Nassar N.N., Hassan A., Pereira-Almao P. (2011). Effect of Surface Acidity and Basicity of Aluminas on Asphaltene Adsorption and Oxidation. J. Colloid Interface Sci..

[27] Simão A., Domínguez-Álvarez E., Yuan C., Suwaid M.A., Varfolomeev M.A., Ancheyta J., Al-mishaal O.F., Kudryashov S.I., Afanasiev I.S., Antonenko D.A. (2022). On the Use of Metallic Nanoparticulated Catalysts for In-Situ Oil Upgrading. Fuel.

[28] Rana M.S., Ancheyta J., Rayo P., Maity S.K. (2004). Effect of Alumina Preparation on Hydrodemetallization and Hydrodesulfurization of Maya Crude. Catal. Today.

[29] Zhukova A.I., Chuklina S.G., Maslenkova S.A. (2021). Study of Cu Modified Zr and Al Mixed Oxides in Ethanol Conversion: The Structure-Catalytic Activity Relationship. Catal. Today.

[30] Bai P., Etim U.J., Yan Z., Mintova S., Zhang Z., Zhong Z., Gao X. (2019). Fluid Catalytic Cracking Technology: Current Status and Recent Discoveries on Catalyst Contamination. Catal. Rev..

[31] Cardona L., Medina O.E., Cespedes S., Lopera S.H., Cortes F.B., Franco C.A. (2021). Effect of Steam Quality on Extra-Heavy Crude Oil Upgrading and Oil Recovery Assisted with PdO and NiO-Functionalized Al_2_O_3_ Nanoparticles. Processes.

[32] Mukhamed’yarova A.N., Gareev B.I., Nurgaliev D.K., Aliev F.A., Vakhin A.V. (2021). A Review on the Role of Amorphous Aluminum Compounds in Catalysis: Avenues of Investigation and Potential Application in Petrochemistry and Oil Refining. Processes.

[33] Abdelsalam Y.I.I., Galiakhmetova L.K., Sharifullin A.V., Tajik A., Mukhamatdinova R.E., Davletshin R.R., Vakhin A.V. (2025). Comparative Study of the Catalytic Effects of Al (CH_3_COO)_3_ and Al_2_ (SO_4_)_3_ on Heavy Oil Aquathermolysis in CO_2_ and N_2_ Atmospheres. Appl. Catal. A Gen..

[34] Maity S.K., Ancheyta J., Marroquín G. (2010). Catalytic Aquathermolysis Used for Viscosity Reduction of Heavy Crude Oils: A Review. Energy Fuels.

[35] Zhang W., Li Q., Li Y., Dong S., Peng S., Chen G. (2022). Viscosity Reduction and Mechanism of Aquathermolysis of Heavy Oil Co-Catalyzed by Bentonite and Transition Metal Complexes. Catalysts.

[36] Zhang Y., Yan J., Li M., Chen X., Zhang L. (2024). The Effect of Free Radical Initiator in Promoting Aquathermolysis of Heavy Oil under Mild Conditions. Fuel.

[37] Avbenake O.P., Al-Hajri R.S., Jibril B.Y. (2019). Catalytic Upgrading of Heavy Oil Using NiCo/γ-Al_2_O_3_ Catalyst: Effect of Initial Atmosphere and Water-Gas Shift Reaction. Fuel.

[38] Zhao F., Xu T., Zhu G., Wang K., Xu X., Liu L. (2022). A Review on the Role of Hydrogen Donors in Upgrading Heavy Oil and Bitumen. Sustain. Energy Fuels.

[39] Djimasbe R., Varfolomeev M.A., Al-Muntaser A.A., Yuan C., Feoktistov D.A., Suwaid M.A., Kirgizov A.J., Davletshin R.R., Zinnatullin A.L., Fatou S.D. (2022). Oil Dispersed Nickel-Based Catalyst for Catalytic Upgrading of Heavy Oil Using Supercritical Water. Fuel.

[40] Shen Z., Fang X., He W., Zhang L., Li Y., Qi G., Xin X., Zhao B., Chen G. (2024). Enhanced Aquathermolysis of Water–Heavy Oil–Ethanol Catalyzed by B@ Zn (II) L at Low Temperature. Molecules.

[41] Demirbas A., Alidrisi H., Balubaid M.A. (2015). API Gravity, Sulfur Content, and Desulfurization of Crude Oil. Pet. Sci. Technol..

[42] Agarwal P., Sharma D.K. (2010). Comparative Studies on the Bio-Desulfurization of Crude Oil with Other Desulfurization Techniques and Deep Desulfurization through Integrated Processes. Energy Fuels.

[43] Gafurov M.R., Volodin M.A., Rodionov A.A., Sorokina A.T., Dolomatov M.Y., Petrov A.V., Vakhin A.V., Mamin G.V., Orlinskii S.B. (2018). EPR Study of Spectra Transformations of the Intrinsic Vanadyl-Porphyrin Complexes in Heavy Crude Oils with Temperature to Probe the Asphaltenes’ Aggregation. J. Pet. Sci. Eng..

[44] Shabalin K.V., Foss L.E., Borisova Y.Y., Borisov D.N., Yakubova S.G., Yakubov M.R. (2020). Study of the Heavy Oil Asphaltenes Oxidation Products Composition Using EPR and IR Spectroscopy. Pet. Sci. Technol..

[45] Chen W.-H., Hsieh T.-C., Jiang T.L. (2008). An Experimental Study on Carbon Monoxide Conversion and Hydrogen Generation from Water Gas Shift Reaction. Energy Convers. Manag..

[46] Chen W.-H., Chen C.-Y. (2020). Water Gas Shift Reaction for Hydrogen Production and Carbon Dioxide Capture: A Review. Appl. Energy.

[47] Mustakimova E.A., Baigildin I.G., Talanova M.Y., Cherednichenko K.A., Maximov A.L., Karakhanov E.A., Vutolkina A.V. (2025). Simultaneous Hydrotransformation of Aromatics and Sulfur Compounds over Unsupported NiMoS Catalysts under Water Gas Shift Reaction Conditions. Fuel.

[48] Aliev F., Abdelsalam Y., Lapuk S., Khelkhal M.A., Suwaid M., Vakhin A. (2024). Efficient Heavy Oil Upgrading with Water-Soluble Nickel and Copper Acetate Catalysts. Ind. Eng. Chem. Res..

